# Multifamily group evaluation with Score 15 questionnaire

**DOI:** 10.1192/j.eurpsy.2024.1471

**Published:** 2024-08-27

**Authors:** B. Gamo Bravo, M. E. Gonzalez Laynez, S. M. Bañon Gonzalez, N. Ogando Portilla

**Affiliations:** ^1^psichiatry, University hospital infanta Sofia, San Sebastian de los Reyes; ^2^psichiatry, University hospital of Toledo, Toledo, Spain

## Abstract

**Introduction:**

The multifamily group that has been underway since April 2019 in Alcobendas, Madrid is described. A group that serves people diagnosed with mental disorder and their families, with the aim of improving their health and quality of life. It is about facilitating and improving the basic communication of relational aspects and healthy bonds. It is intended to offer a space where you can think together about the experiences lived in your own family with the rest of the group

**Objectives:**

Assess the evolution and improvement of the patient and family members with the Score 15 questionnaire, The Score is a way of giving users a voice about the therapy process, not about the contents of their problems, but about their perception of the effectiveness of therapeutic work and for professionals it is an opportunity to obtain important feedback from their work.

**Methods:**

Using the Score !5 questionnaire on all participants in the group at time zero and after 12 sessions

**Results:**

Improvement in the family description items, and in the quantitative improvement in scoring of the following questions: What degree of severity would indicate? Do you think therapy will be helpful/has it been helpful to you?

**Conclusions:**

Family therapy in the modality of Multifamily Groups provides an improvement in intrafamily communication, its links and therefore in the rest of social communication, facilitating the exit from loneliness and misunderstanding and in turn broadens the understanding and understanding by therapists

**Disclosure of Interest:**

None Declared

